# Incidence and mortality of hypotension in the emergency department; an 12-year population based study

**DOI:** 10.1186/1757-7241-23-S1-A22

**Published:** 2015-07-16

**Authors:** Jon G Holler, Daniel P Henriksen, Søren Mikkelsen, Court Pedersen, Annmarie Lassen

**Affiliations:** 1 Department of Emergency Medicine, OUH Odense University Hospital, Odense, Denmark; 2 Department of Clinical Chemistry & Pharmacology, OUH Odense University Hospital, Odense, Denmark; 3 Department of Anesthesiology and Intensive Care Medicine, OUH Odense University Hospital, Odense, Denmark; 4 Department of Infectious Diseases, OUH Odense University Hospital, Odense, Denmark

## Background

One of the symptoms of shock is hypotension. Incidence and mortality of unselected patients with hypotension in the Emergency Department (ED) is unclarified. The aim was to describe the incidence rates and overall mortality of hypotensive patients in the ED in an 12-year period.

## Methods

We identified all patients aged ≥ 18 years with a first time presentation to the ED with hypotension within the study period. Patients were included if their initial systolic blood pressure (SBP) recording was below 100 mmHg upon arrival to the ED of Odense University Hospital, Denmark, during the study period (1st January 2000 to 31st December 2011). We excluded patients if they did not have a valid unique personal identification number or lived outside the hospital's catchment-area. The study population was linked to several population-based registers using the unique Danish personal identification number in order to determine comorbidity and overall mortality proportions. Incidence rates (IRs) were calculated per 100,000 person years at risk (pyar) and presented as crude annual rates.

## Results

We identified 3,378 cases with a median age of 68 years (IQR 51-80); 1,716 (50.8%) were men, and 872 (25.1%) had Charlson Comorbidity Index > 2. The median SBP was 91 mmHg (IQR 84-96). The IRs of hypotension ranged from 124/100,000 pyar (95% CI: 109-139) in 2000 to 174/100,000 pyar (95% CI: 158-192) in 2011. The IRs showed a stationary trend between 2000-2008 (Median IR; 128/100,000 pyar), but increased during the years 2009-2011 (Median IR; 164/100,000 pyar). The IRs according to age groups showed an increasing trend with 50/100,000 pyar (95% CI: 45-55) among age 18-39 years to 1028/100,000 pyar (95% CI: 952-1109) among age 85+ years. The overall 7-day and 90-day mortality was 521 (15.4%) and 966 (28.6%) respectively with a stable trend throughout the period. Highest 90-day mortality was 518 (38.1%) among age 65-84 years compared to lowest mortality of 20 (4.2%) among 18-39 years during the period.

## Conclusion

During the period 2000-2011 the crude incidence of hypotension have increased, especially during the years 2009-2011 with increasing incidence rates among the elderly. The overall 7-day and 90-day mortality was 15.4% and 28.6% respectively.

